# Evaluation of drought and salinity tolerance potentials of different soybean genotypes based upon physiological, biochemical, and genetic indicators

**DOI:** 10.3389/fpls.2024.1466363

**Published:** 2024-12-06

**Authors:** Yahya Alzahrani

**Affiliations:** Department of Biological Sciences, Faculty of Science, King Abdulaziz University, Jeddah, Saudi Arabia

**Keywords:** antioxidants, oxidative stress, integrated response, water relations, gene expression

## Abstract

The present study has evaluated different soybean genotypes to understand the salt and drought tolerance mechanisms based on physiological traits (photosynthesis, stomatal conductance, chlorophyll, and cell membrane stability), antioxidant enzymes (superoxide dismutase, catalase, and peroxidase), reactive oxygen species (H_2_O_2_ and O_2_^•−^), osmolytes (glycine betaine, proline, and Na^+^/K^+^), plant water relations (relative water content, water potential, and solute potential) and expression of related genes (*GmCAT1*, *GmPOD1*, *GmSOD*, *GmP5CS*, *GmNHX1*, *GmAKT1*, *GmDREB1*, and *GmARF1*). The experiment was conducted in a two-factorial arrangement using randomized complete block design (RCBD) with genotypes as one factor and salt, drought, and control treatments as the other factor. All physiological traits, relative water content, and water potential decreased significantly in all soybean genotypes due to individual and combined treatments of drought and salt stress, with significantly less decrease in soybean genotypes G4620RX, DM45X61, and NARC-21. Besides that, the activity of antioxidant enzymes, production of ROS, accumulation of osmolytes, solute potential, and Na^+^/K^+^ ratio were increased significantly in all soybean genotypes under salt and water deficit conditions. As a whole, the soybean genotypes G4620RX, DM45X61, and NARC-21 showed the maximum enzymatic activity with less increase in ROS and Na^+^/K^+^ in addition to a high accumulation of osmolytes and an increase in solute potential. Correspondingly, the genotypes exhibiting high physiological and biochemical tolerance to drought and salt stresses showed the high expression of genes imparting the stress tolerance. Moreover, correlation, heatmap, and principal component analysis further confirmed the varying physiological and biochemical responses of all soybean genotypes under individual and combined applications of drought and salinity stresses. Overall, the present study confirmed that plants opt for the integrated physiological, biochemical, and genetic approaches to counteract the harmful effects of environmental stresses.

## 1 Introduction

Soybean is a globally important leguminous crop, famous for its rich oil and protein contents. This crop is ecologically important and has a tendency to improve the soil’s fertility due to the assistance of symbiotically associated bacteria. In terms of cultivated area, soybean comes at fourth place after wheat, rice, and maize and at first place among legumes (Sedibe et al., 2023). In the year 2020, it was cultivated on a 127-million-hectare area of the world, with an annual yield of 354 million tonnes (Staniak et al., 2023). The yield of soybean varies from year to year due to various sorts of environmental factors such as drought, heat, and salinity (Wójcik-Gront et al., 2022). The stress conditions, particularly drought and salinity, induce disturbances at the cellular level causing secondary stresses, e.g., oxidative stress, owing to the generation of reactive oxygen species (ROS) (Hasanuzzaman et al., 2020). Besides that, ROS damage the biochemical structure of cell membranes due to lipid peroxidation and protein denaturation (Feng et al., 2023). Plants are naturally equipped with an antioxidant system to scavenge the ROS (Khan et al., 2009). In this regard, a speedy change in the activities of antioxidant enzymes such as superoxide dismutase (SOD), peroxidase (POD), and catalase (CAT) is an indicator of plant tolerance to oxidative stress (Wang et al., 2018). Furthermore, the abiotic stresses interrupt the physiological processes (photosynthesis) due to the damage of enzymes involved in chlorophyll synthesis (Rajput et al., 2021). Like all living organisms, plants also have a tendency to counter the impacts of stress through different types of homeostatic mechanisms. In this context, plant increases the production of osmoprotectants like proline and glycine betaine (Wani et al., 2013; Xu et al., 2023). Besides that, the water status of plant changes due to abiotic stresses that further change the water potential and solute potential. Therefore, plants opt for some osmotic adjustments by producing some osmolytes (Wijewardana et al., 2019; Otie et al., 2021). These osmolytes play adaptive roles in plants in facilitating the osmotic adjustments and safeguarding the sub-cellular structures in stressed plants (Ding et al., 2024). On the other hand, salinity stress increases the accumulation of Na^+^ in plant cells (Jin et al., 2022). However, like all other living organisms, plants also respond, and as a homeostatic adjustment, plants enhance the efflux of Na^+^ due to the influx of K^+^ (Sun et al., 2021). It is essential for a plant to maintain a low Na^+^/K^+^ ratio under saline conditions to carry out normal physiological and biochemical processes (Sun et al., 2019). Furthermore, soybean is an ideal system to understand the genetic dynamics of drought and salinity tolerance in plants. Plants’ tolerance to drought and salinity is a complex trait regulated by various genes; therefore, it is important to understand various regulatory mechanisms in association with the expression of genes involved in regulatory networks—for instance, under oxidative stress, the overexpressed *GmSOD1*, *GmCAT1*, and *GmPOD1* enhance the activities of SOD, CAT, and POD, respectively, that accelerate the detoxification of ROS such as H_2_O_2_ and O_2_^.-^ (Li et al., 2020; Xu et al., 2023). The enzyme pyrroline-5-carboxylate synthase is involved in the synthesis of pyrroline-5-carboxylate (P5CS), a key precursor in proline synthesis exhibiting a protective role during drought and salinity stress (Xu et al., 2023). Besides that, the sodium and proton exchanger (NHX) mediates the efflux of Na^+^ through regulating the expression of genes, while *AKT1* triggers the influx of K^+^ to balance the Na^+^/K^+^ ratio under saline conditions (Sun et al., 2019; Wang et al., 2021). Furthermore, Sun et al. (2019) have found that apart from regulating the efflux of Na^+^, *GmNHX1* regulates the expression of a series of other genes including *SKOR*, *SOS1*, and *AKT1* involved in salinity tolerance. Moreover, Wang et al. (2021) reported the essential role of *GmAKT1* in the uptake of K^+^ to balance the Na^+^/K^+^ ratio under saline conditions. Furthermore, drought stress triggers the expression of dehydration-responsive element binding protein (*DREB*) that regulates the expression of other genes imparting drought tolerance in soybean (Zhou et al., 2022). The auxin-responsive factors (*ARF*) are also responsive to drought stress and regulate biochemical processes to enhance the plants’ drought tolerance potential (Ha et al., 2015). To date, various studies have been conducted to investigate the potential impact of drought and salt stress on soybean based on physiological and biochemical indicators; however, limited knowledge is available on physio-chemical changes, oxidative stress, osmolytic dynamics, and respective genetic control. The present study intended to elucidate the impacts of individual and combined treatments of drought and salt stress on the physiological, biochemical, and genetic indicators of stress tolerance in different soybean cultivars for a comparative understanding of the dynamics of stress tolerance.

## 2 Materials and methods

The present study was conducted in a glass house located at the experimental site of King Abdulaziz University, Jeddah, Saudi Arabia, 21°32′36″ N and 39°10′22″ E, at 12 m above sea level. Six different soybean cultivars—Rawal-1, Swat-84, NARC-1, and NARC-21 collected from National Agricultural Research Center (NARC), Islamabad, Pakistan, and G4620RX and DM45X61 collected from USA—were evaluated for salinity and drought tolerance. The tri-replicate experiment was conducted in a randomized complete block design (RCBD) using factorial arrangements with soybean cultivars as one factor and with drought and salinity treatments as second factor.

### 2.1 Plant husbandry and treatments

Seeds were sown in a plastic container with 60-cm height and 35-cm diameter at a depth of 2.5 cm. The pots were filled with 1:3 mixtures of soil and vermiculite. The conditions within the growth chamber were optimized following the procedure of Liu et al. (2017) at relative humidity of 75%, day/night temperature of 28°C/20°C, photoperiod of 16 h, and irradiance of 240 μmol m^-2^ s^-1^. The pots were watered to full capacity until the second-node stage (V2). Afterward, the irrigation water was supplemented with 15% (m/v) polyethylene glycol (PEG-6000) to induce drought stress and supplemented with 150 mM NaCl to induce salt stress. The drought treatment was applied using 300 mL PEG solution (Hamayun et al., 2010), while salt treatment was applied using 300 mL of NaCl solution (Hasanuzzaman et al., 2022). Besides that, the control set of plants received normal water as per requirement. When the plants attained the third vegetative stage with three nodes and axillary buds, two to three leaves from the upper side were taken from the stressed and control plants. These leaves were kept in liquid nitrogen and stored at -80°C before RNA extraction and assessment of enzymatic activity. Furthermore, for each treatment in a replicate, four pots each with three plants were used.

### 2.2 Physiological analysis

The chlorophyll content was estimated with the help of SPAD-502plus apparatus, while photosynthesis and stomatal conductance rates were estimated using a special apparatus, IRGA apparatus (ADC Bioscientific, UK). The cell membrane stability percentage (CMSP) was calculated by recording the leakage of electrolyte from leaves under applied treatments using the relative conductivity method (Ibrahim and Quick, 2001). For the estimation of physiological traits, five plants from each treatment were selected for data collection. The data were averaged before subjecting to statistical analysis. Besides that, the results depicting a significant variation at the third vegetative stage with three nodes and axillary buds were included in the analysis.

### 2.3 Analysis of biochemical traits

#### 2.3.1 Osmolytic analysis

Among biochemical parameters, proline content was determined with the help of a UV–Vis spectrophotometer (N5000, Shanghai, China) based upon its reactivity with ninhydrin (Carillo and Gibon, 2011), while glycine betaine (GB) was determined using HPLC (Shimadzu Corp, Japan) following the method described by Ma et al. (2007). On the other hand, the Na^+^ and K^+^ content from soybean leaves was estimated following the protocol described by Havre (1961). For this purpose, the oven-dried leaf samples were crushed in the form of fine powder, and 0.5 g of each sample was mixed in a mixture of 8 mL HNO_3_ and 3 mL HClO_4_ and kept at room temperature for 12 h. Afterward, the mixture was burnt at 300°C for 3 h using a flame photometer (Sigma-Aldrich, USA). Subsequently, distilled water was added in the burnt sample to attain a final volume of 50 mL. Finally, the concentration of Na^+^ and K^+^, respectively, was calculated using a flame photometer (Sigma-Aldrich, USA), and the Na^+^/K^+^ ratio was calculated. For the quantification of biochemical traits, five plants from each treatment were selected for data collection on average basis. Besides that, the results depicting a significant variation at the third vegetative stage with three nodes and axillary buds were included in the analysis.

#### 2.3.2 Estimation of enzymatic activity and reactive oxygen species

For the assessment of catalytic activity of antioxidant enzymes (SOD, POD, and CAT), the frozen leaf sample weighing 1.5 g was thoroughly mixed in 1.5 mL of 0.1 M ice-cold Tris-HCl having a pH of 7.4. Subsequently, the mixture was centrifuged at 20,000 *g* for 15 to 20 min at 4°C. Afterward, the supernatant was isolated, and enzymatic activity was recorded following the protocols described by Alici and Arabaci (2016). The enzymatic activity of the catalase enzyme was estimated spectrophotometrically at room temperature using H_2_O_2_ as substrate and recording absorbance reduction at 240 nm. Similarly, the enzymatic activity of peroxidase was recorded by employing a spectrophotometer using 4-methylcatechol as substrate at an absorption standard of 420 nm. Moreover, the activity of superoxide dismutase was estimated based on its ability to inhibit the photoreduction of nitroblue-tetrazolium against the standard absorption curve of 560 nm. The enzymatic activities of all enzymes were measured in enzyme units (U mg-^1^ of protein). The H_2_O_2_ and superoxide contents were determined following the procedure opted by Ba et al. (2013). The O_2_^•−^ content was determined following the protocol described by Yang et al. (2020). For this purpose, 0.5 g flag leaf samples were homogenously mixed in phosphate buffer (65 mM with pH 7.8) and subjected to centrifugation for 10 min at 4°C and 5,000 × *g*. Subsequently, the supernatant was extracted and incubated for 20 min at 25°C in a mixture of phosphate buffer and 10 mM hydroxylamine chlorhydrate. After incubation, 17 mM sulfanilamide and 7 mM α-naphthylamine were added in the mixture and incubated for a further 20 min. Besides that, the formation rate of O_2_^•−^ was recorded using a spectrophotometer at absorbance of 530 nm from the standard curve of NaNO_2_. On the other hand, H_2_O_2_ was determined following the procedure described by Jun et al. (2000). For this purpose, the absorbance was recorded at 410 nm, and the H_2_O_2_ content of leaves was measured from H_2_O_2_ solution-derived standard curve.

### 2.4 Determination of water-related attributes

The procedure adopted by Bannister (1964) was used to estimate the relative water content (RWC) using the formula


RWC=½(Fresh Weight–Dry Weight)/(Total Weight–Dry Weight)×100

Besides that, the water potential (ψ_w_) and osmotic potential (ψ_s_) were measured according to the method explained by Turner (1981). The value of ψ_w_ was estimated using Scholander bomb (PMS Instrument Company, Albany, USA), following the methodology given by the manufacturer. On the other hand, the ψ_s_ osmotic potential (ψs) was estimated using an osmometer (Advanced Instruments, Norwood, USA), according to the instructions provided.

### 2.5 Gene expression analysis

The genes associated with abiotic stress tolerance such as *GmCAT1*, *GmPOD1*, *GmSOD*, *GmP5CS*, *GmNHX1*, and *GmAKT1* were analyzed for relative expression. The leaf samples collected for RNA extraction were stored at -80°C sharp after collection. RNA was extracted following the standard procedure of the manufacturer using RNeasy kit (Qiagen, Germany). The cDNA library was constructed using QuantiTect reverse transcription kit (Qiagen, Germany) from 2 µg RNA. Furthermore, qRT-PCR (QR0200, Sigma Aldrich, USA) was executed using SYBER Green Kit (Sigma-Aldrich, USA), while *GmActin* was used to normalize the gene expression. Likewise with Ahmed et al. (2022), three technical and three biological replicates were used for the analysis and confirmation of each expression profile. Moreover, double delta Ct value was used to calculate the relative gene expression of each sample. The primers used in the study are indicated in **Table 1**.

**Table 1:** List of gene primers used in the qRT-PCR analysis.

Gene	Primer	Reference
*GmCAT1*	ACTACAAATTCTGG TGCTCCTA (F) TGCAAGCTTCTCC ACAAGA (R)	Li et al., 2020
*GmPOD1*	GCTTTGAGCACCAT TAGA (F) TTGGTGAAGGGTC TAGTA (R)	Li et al., 2020
*GmSOD*	TGGTCTCCATGGCT TCCAT (F) GCTAACGGTACCA TCATCA (R)	Li et al., 2020
*GmP5CS*	CGAACTGAGCTTGCAGAGGGGC (F) TCGCTTAGCCTCCTTGCCTCC (R)	Xu et al., 2023
*GmNHX1*	AAGCAGCATCCGTGCTTTAC (F) CCTGCCACCAAAAACAGGAC (R)	Sun et al., 2019
*GmAKT1*	AAAGGTCTCACTCATCAACAACGA (F) TCGGCAAAAGAGGCAAAATAAG (R)	Wang et al., 2021
*GmDREB1*	CGATGAAACCTTACCGTGGAA (F) AAGTCGGGCTTGAGATTGAG (R)	Zhou et al., 2022
*GmARF1*	CATAGGCAGCTAGATTTTGCG (F) AATTACTAAGGTGCCTCCAAGG (R)	Ha et al., 2015
*Actin*	ATGGCTGATGGTGAAGACATTC (F) TCCATGCTCAATAGGGTACTTG (R)	Sun et al., 2019

### 2.6 Statistical analysis

The data were subjected to analysis of variance (ANOVA) at a probability level of 5% using the computer-based program Statistix8.1. (McGraw-Hill, 2008). The R-packages (RStudio Team, 2020) “factoextra” and “FactoMineR” were used for the principal component analysis (PCA). Pearson’s correlation was performed by using the R package “GGally”, and heatmap was constructed by using the R package “pheatmap”.

## 3 Results

### 3.1 Physiological outcomes

**Figure 1:**
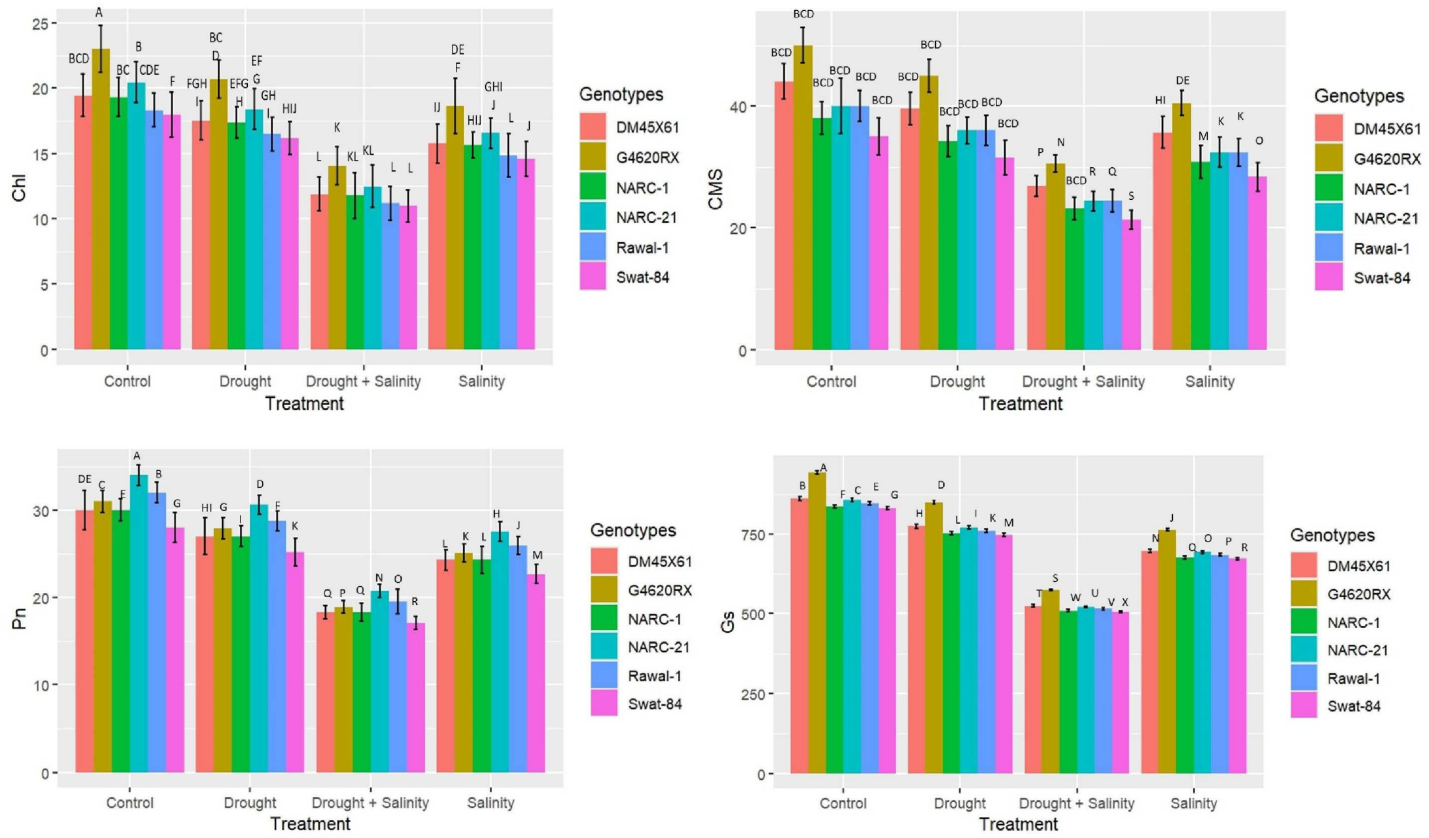
Effect of individual and combined applications of drought and salt stress on the physiological traits (photosynthesis, Pn; stomatal conductance, Gs; chlorophyll, Chl; cell membrane stability, CMS) of soybean genotypes. The values indicated in the figure indicate mean estimates analyzed during a tri-replicate two-factorial experiment at *p* ≤ 0.05. Units: Pn (µmol m^-2^ s^-1^), Gs (mmol m^-2^ s^-1^), Chl (g kg^-1^), CMS (%). The asterisk indicates that the bar values following different letters are significantly different at *p* ≤ 0.05.

All physiological traits such as chlorophyll (chl), photosynthesis (Pn), stomatal conductance (Gs), and cell membrane stability (CMS) varied significantly (*p* ≤ 0.05) due to the individual and combined effects of drought and salinity treatments in all soybean cultivars (**Figure 1**). All soybean genotypes illustrated a significant reduction in all physiological traits under split and integrated applications of drought and salinity stress; however, this reduction was significantly higher under the integrated application of drought and salinity stress compared with split applications (**Figure 1**). However, the individual application of drought and salinity did not show a significant difference in the reduction of corresponding physiological traits compared with each other. Besides that, among genotypes, G4620RX recorded the minimum decrease in chl (13 g kg^-1^), Gs (600 mmol m^-2^ s^-1^), and CMS (30%), followed by NARC-21, Rawal-1, DMX4561, NARC-1, and Swat-84, respectively (**Figure 1**) at the combined application of drought and salt stress. However, Pn illustrated a minimum decrease in NARC-1 (20 µmol m^-2^ s^-1^) followed by G4620RX, Rawal-1, DMX4561, NARC-21, and Swat-84, respectively, at the integrated treatments of drought and salt stress.

### 3.2 Biochemical traits

**Figure 2:**
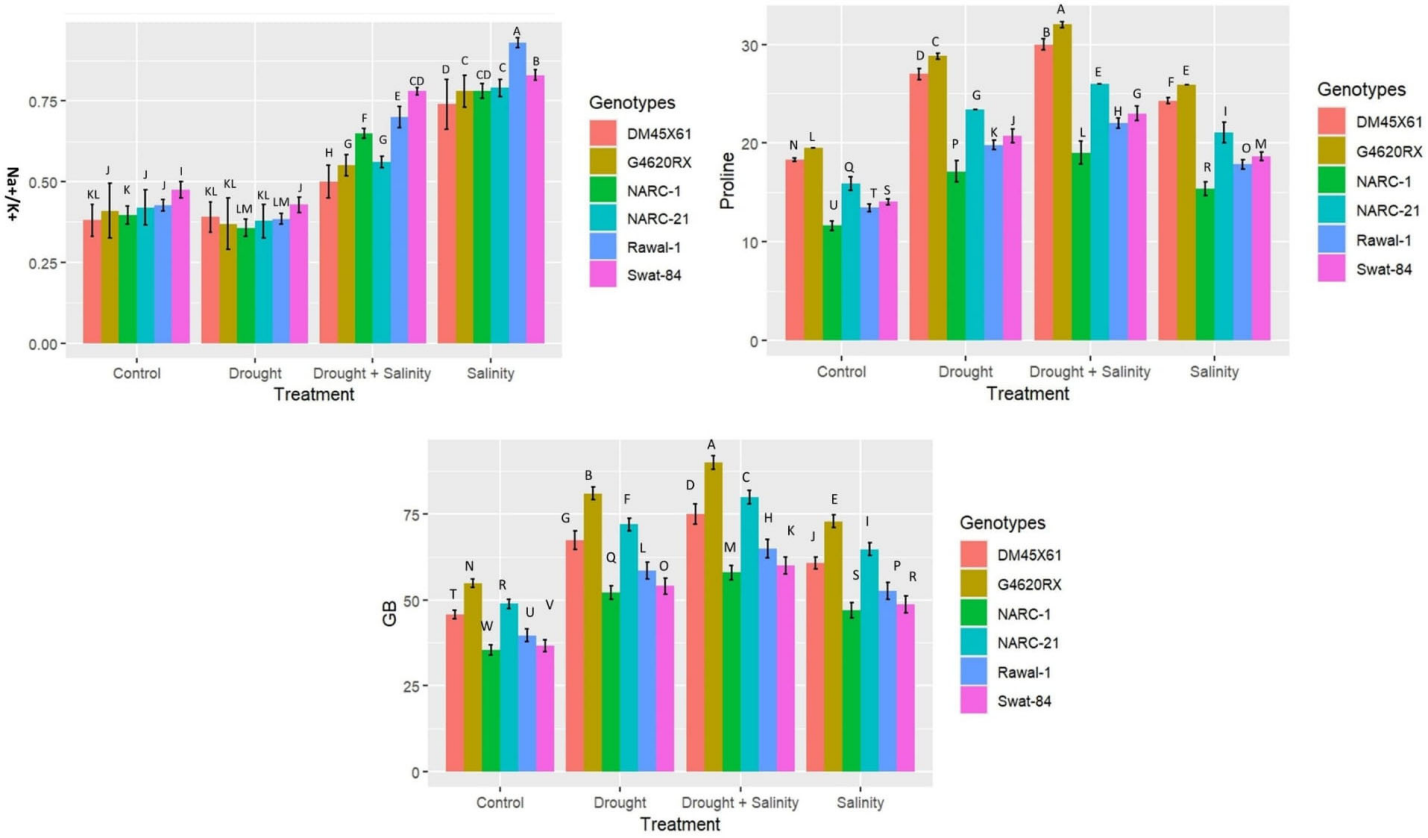
Effect of individual and combined treatments of drought and salinity stress on osmolytes (glycine betaine, GB; proline) and Na^+^/K^+^ in soybean genotypes. The values indicated in the figure are means analyzed during a tri-replicate two-factorial experiment at *p* ≤ 0.05. Units: GB (µg g^-1^FW), proline (mg g^–1^ FW). The asterisk indicates that the bar values following different letters are significantly different at *p* ≤ 0.05.

All biochemical traits including proline, glycine betaine (GB), and Na^+^/K^+^ illustrated a statistically significant (*p* ≤ 0.05) variation under individual and combined applications of drought and salinity treatments compared with the control treatment (**Figure 2**). All soybean genotypes exhibited a significant (*p* ≤ 0.05) increase in the level of the osmolytes proline and GB under the isolated and combined application of drought and salinity; however, this increase was more dramatic under combined application compared with individual application (**Figure 2**). The Na^+^/K^+^ ratio increased significantly (*p* ≤ 0.05) in all soybean genotypes due to the individual application of salinity stress followed by the combined application, control treatment, and drought stress (**Figure 2**). Besides that, among genotypes, G4620RX showed the maximum increase in osmolytes (proline = 38 mg g^–1^ FW, GB = 86 µg g^-1^FW), followed by DMX4561, NARC-21, Swat-84, Rawal-1, and NARC-1 (**Figure 2**) at the combined treatments of salt and water deficit treatments. On the other hand, the soybean genotype Swat-84 (0.80), followed by Rawal-1 and NARC-1, depicted a significant rise in Na^+^/K^+^ ratio under the combined application of drought and salinity stress.

### 3.3 Antioxidant enzymes and ROS

**Figure 3:**
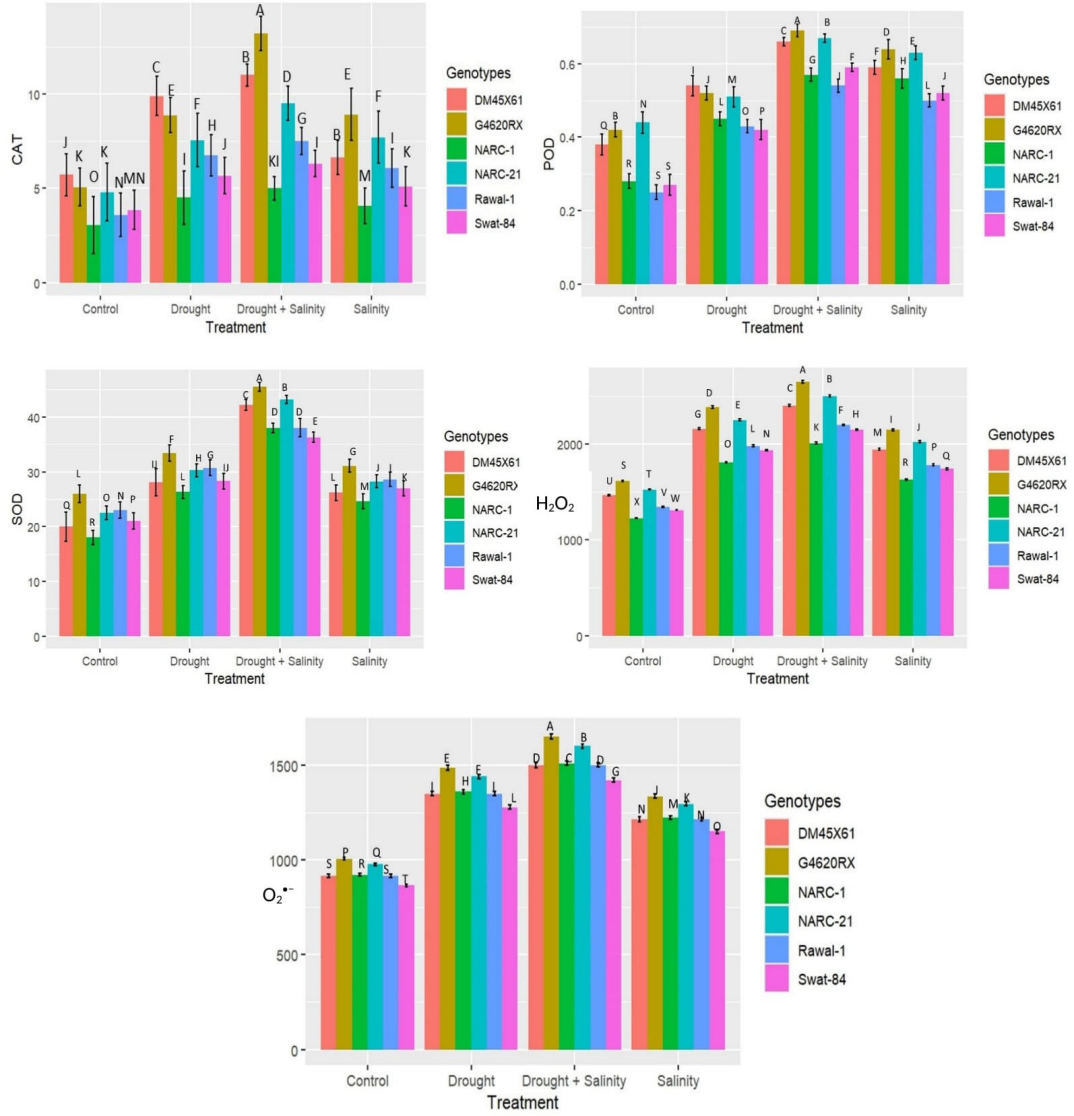
Effect of individual and combined applications of drought and salt stress on antioxidant enzymes (catalase, CAT; peroxidase, POD; superoxide dismutase, SOD) and ROS (H_2_O_2_ and O_2_^•−^) in soybean genotypes. The values indicated in the figure are means analyzed during a tri-replicate two-factorial experiment at *p* ≤ 0.05. Units: Enzymatic activity (U mg^-1^ protein), ROS (nmol µg^-1^FW). The asterisk indicates that the bar values following different letters are significantly different at *p* ≤ 0.05.

Parallel to the production of ROS, H_2_O_2_, and O_2_^•−^, the antioxidant enzymes SOD, CAT, and POD illustrated a significant (*p* ≤ 0.05) increase in catalytic activity in terms of enzyme units under both individual and combined applications of drought and salinity as indicated in **Figure 3**. The combined treatment of drought and salinity revealed a more significant (*p* ≤ 0.05) rise in ROS and enzymatic activity compared with the individual application of stresses (**Figure 3**). Furthermore, among genotypes, G4620RX, followed by DMX4561, NARC-21, Swat-84, Rawal-1, and NARC-1, depicted a statistically significant (*p* ≤ 0.05) difference in ROS production and enzymatic activities compared with the other genotypes under isolated and integrated applications of drought and salinity stress. The genotype G4620RX showed the highest activities of SOD (46 U mg^-1^ protein), POD (0.7 U mg^-1^ protein), and CAT (16 U mg^-1^ protein) along with the highest generation of H_2_O_2_ (2,600 nmol µg^-1^ FW) and O_2_^•−^(1,600 nmol µg^-1^ FW) at the combined treatment of drought and saline stress.

### 3.4 Plant water attributes

**Figure 4:**
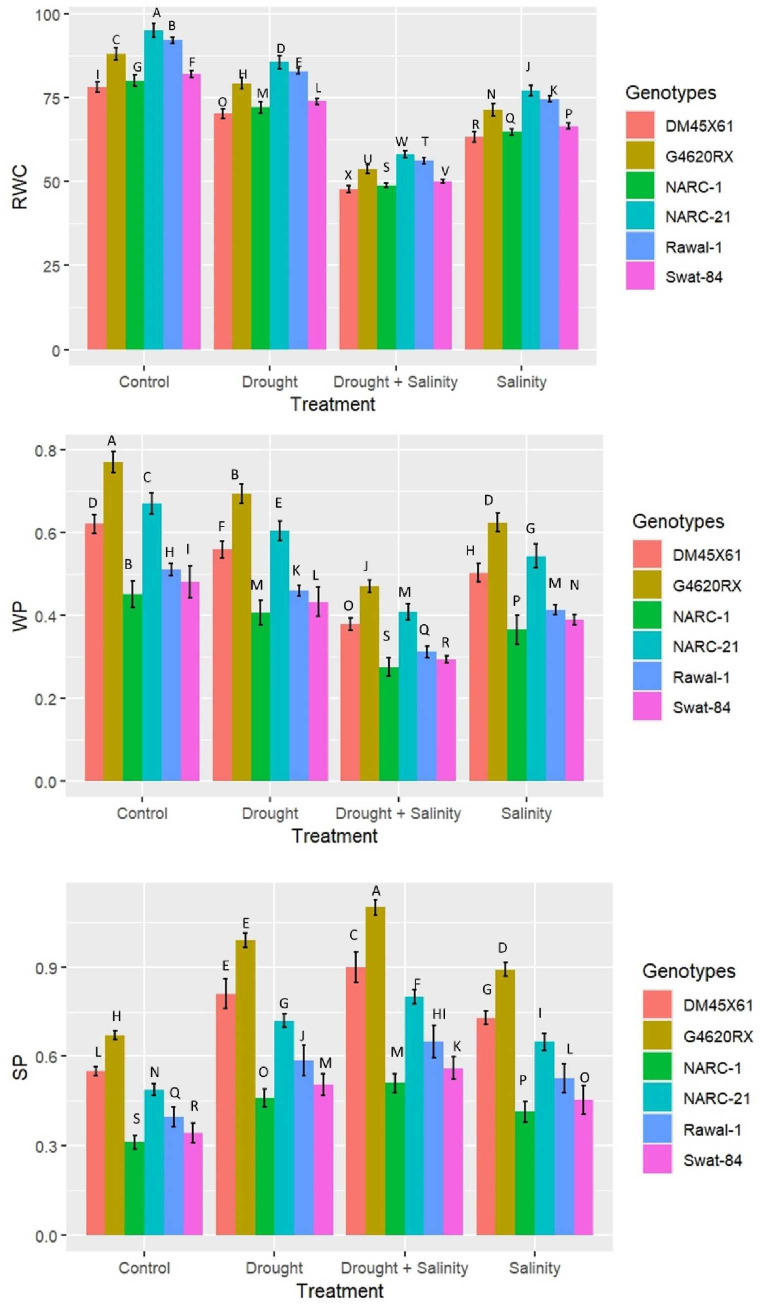
Effect of individual and combined applications of drought and salt stress on water traits (relative water content, RWC; water potential, WP (ψ_w_); osmotic potential, SP (ψ_s_)) of soybean genotypes. The values indicated in the figure indicate mean estimates analyzed during a tri-replicate two-factorial experiment at *p* ≤ 0.05. Units: RWC (%), ψ_w_ (-MPa), ψ_s_ (MPa). The asterisk indicates that the bar values following different letters are significantly different at *p* ≤ 0.05.

The plant water attributes relative water content (RWC), water potential (ψ_w_), and solute potential (ψ_s_) varied significantly (*p* ≤ 0.05) under the individual and combined applications of drought and salinity stresses in all soybean genotypes (**Figure 4**). The RWC and water potential (ψ_w_) decreased significantly (*p* ≤ 0.05) under both split and integrated applications of stresses; however, this decrease was far greater at combined application compared with the individual application of stress (**Figure 4**). Furthermore, among genotypes, G4620RX illustrated a comparatively less reduction in RWC, and water potential (ψ_w_) showed a comparatively less decrease in genotypes NARC-21 (RWC = 55%, ψ_w_ = -0.5 MPa), followed by NARC-21 DMX4561, Rawal-1, and Swat-84, at the combined treatment of drought and salinity (**Figure 4**). Contrary to RWC and water potential (ψ_w_), solute potential (ψ_s_) depicted a significant (*p* ≤ 0.05) increase under the individual and combined versions of drought and salinity stresses with a comparatively high increase at the combined treatment compared with the individual treatments (**Figure 4**). Among genotypes, G4620RX (1.10 MPa), followed by DM45X61, NARC-21, Rawal-1, Swat-84, and NARC-1, showed a significantly high increase in solute potential under the combined treatment of drought and salinity stresses.

### 3.5 Correlation, PCA, and heatmap

**Figure 5:**
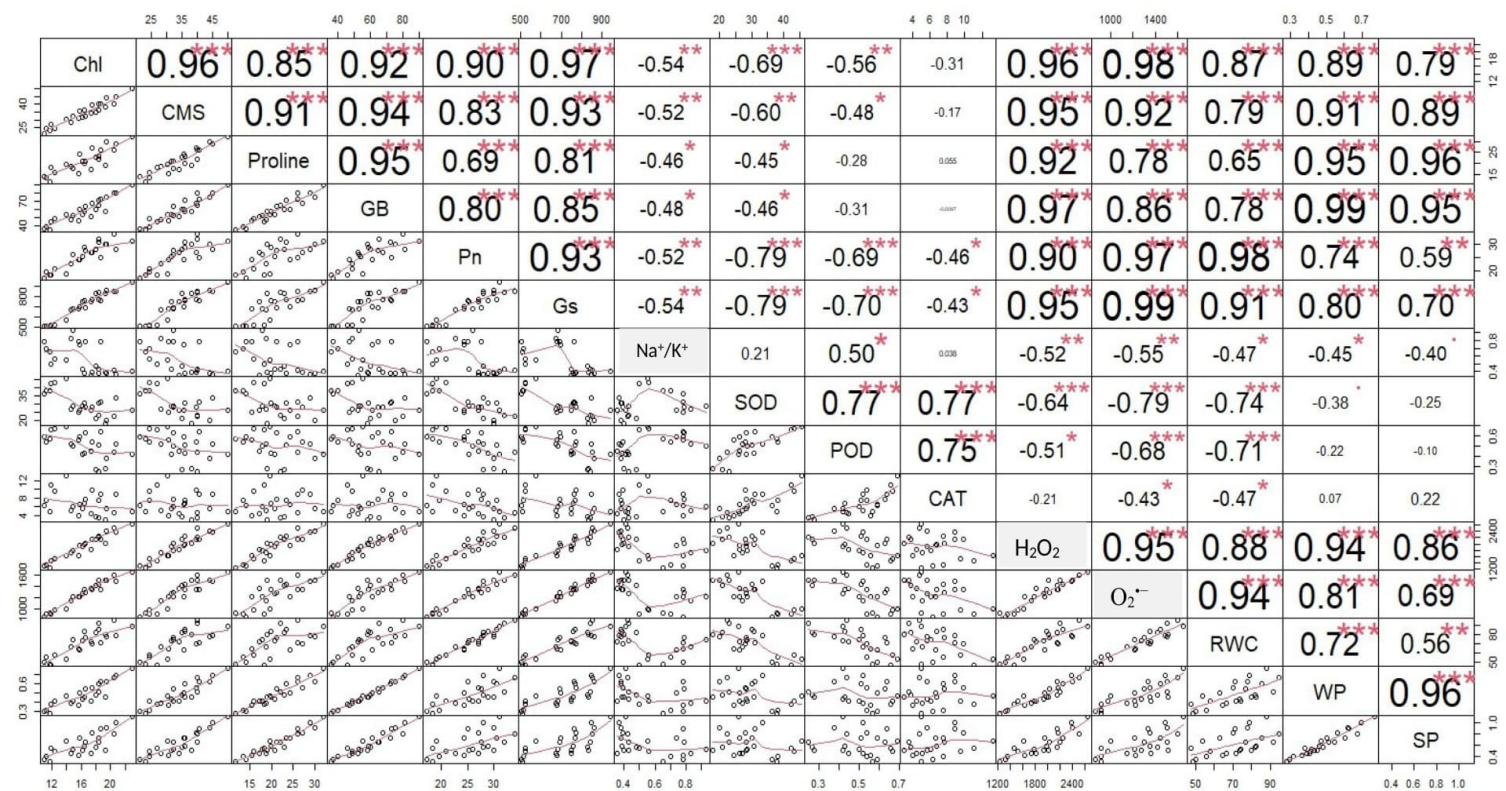
Correlation chart indicating the pairwise association among physiological traits, antioxidant enzymes, reactive oxygen species, osmolytes, and plant water relations in soybean genotypes. The significance of association is proportional to the size of the font in the table. The large font size represents high significance, and the small font size represents no significance. Photosynthesis, Pn; stomatal conductance, Gs; chlorophyll, Chl; cell membrane stability, CMS; catalase, CAT; peroxidase, POD; superoxide dismutase, SOD; glycine betaine, GB; relative water content, RWC; water potential (ψ_w_), WP; osmotic potential (ψ_s_), SP. ***, significant at *p* ≤ 0.001; **, significant at *p* ≤ 0.01; *, significant at *p* ≤ 0.05.

**Figure 6:**
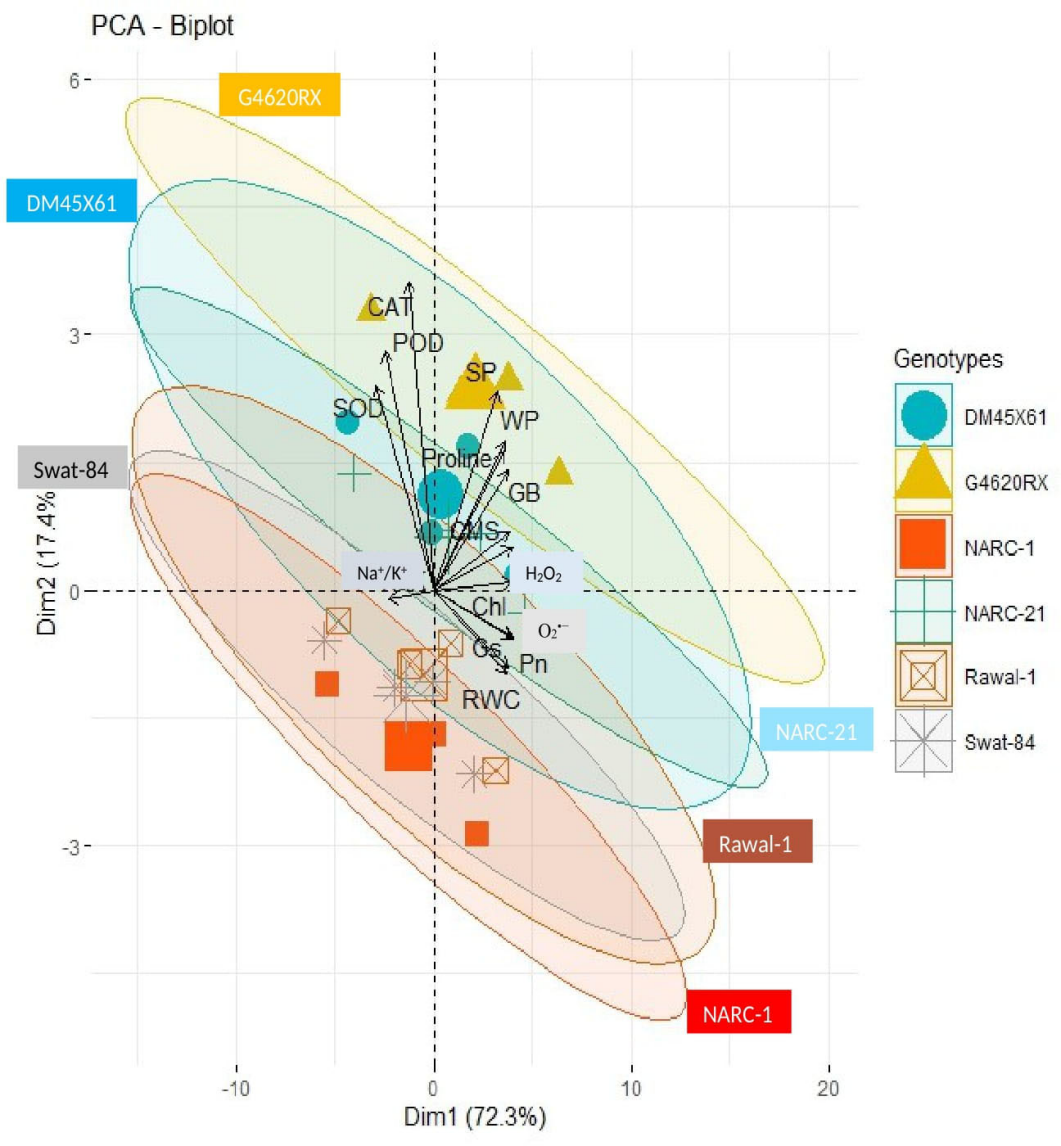
PCA biplot indicating varying degrees of association of physiological traits, antioxidant enzymes, reactive oxygen species, osmolytes, and plant water relation in soybean genotypes. The soybean genotypes (inscribed by eclipses) positioned closer to the traits’ vectors have a stronger association of respective traits compared to genotypes positioned away from the traits’ vectors. Besides that, closer vectors represent the strong association of respective traits and *vice versa*. Photosynthesis, Pn; stomatal conductance, Gs; chlorophyll, Chl; cell membrane stability, CMS; catalase, CAT; peroxidase, POD; superoxide dismutase, SOD; glycine betaine, GB; relative water content, RWC; water potential (ψ_w_), WP; osmotic potential (ψ_s_), SP.

**Figure 7:**
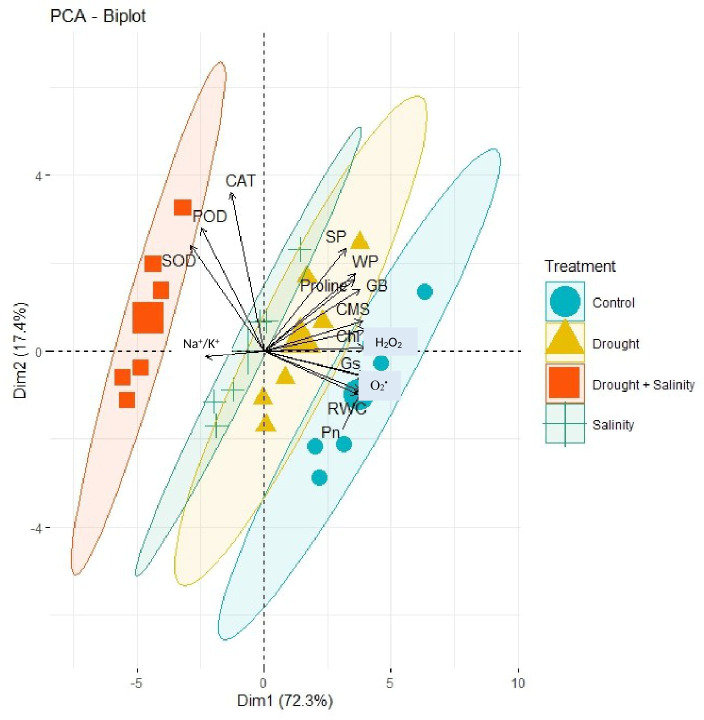
PCA biplot indicating varying degrees of association of physiological traits, antioxidant enzymes, reactive oxygen species, osmolytes, and plant water relation due to the individual and combined effects of drought and salt stress. The changing stresses (inscribed by eclipses) affect the paired association of traits in a different way as indicated by the positioning of stress eclipses with respect to the traits’ vectors. Besides that, closer vectors represent the strong association of respective traits and *vice versa*. Photosynthesis, Pn; stomatal conductance, Gs; chlorophyll, Chl; cell membrane stability, CMS; catalase, CAT; peroxidase, POD; superoxide dismutase, SOD; glycine betaine, GB; relative water content, RWC; water potential (ψ_w_), WP; osmotic potential (ψ_s_), SP.

**Figure 8:**
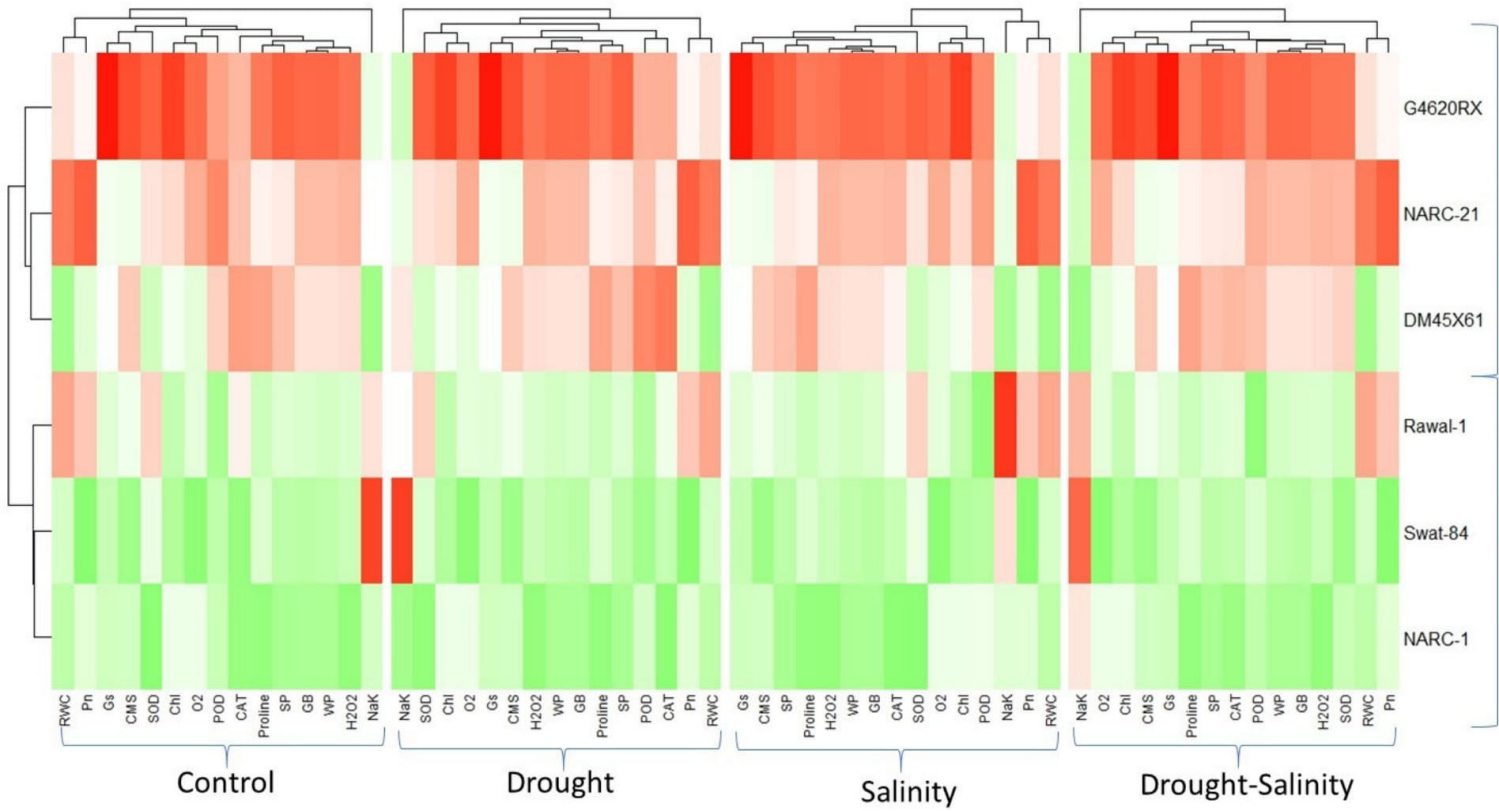
Heatmap categorizing the wheat genotypes in terms of the varied expression of traits in soybean genotypes under individual and combined applications of drought and salt stresses. The varying color pattern of bands (light to dark) illustrates the extent of variation of trait expression in each soybean genotype. Photosynthesis, Pn; stomatal conductance, Gs; chlorophyll, Chl; cell membrane stability, CMS; catalase, CAT; peroxidase, POD; superoxide dismutase, SOD; glycine betaine, GB; relative water content, RWC; water potential (ψ_w_), WP; osmotic potential (ψ_s_), SP. The asterisk indicates that the white color represents no effect, the color change from light to dark green represents minimum to maximum decline in trait expression, and the color change from light to dark red represents minimum to maximum increase in trait expression.

The correlation analysis revealed a significant extent of paired association among physiological traits, osmolytes, ROS, antioxidant enzymes, and plant water relations in both the negative and positive directions (**Figure 5**). Chlorophyll content illustrated a significantly positive paired association with osmolytes (proline and GB), CMS, plant water relations (RWC, ψ_w_, and ψ_s_), Pn, and Gs. On the other hand, chl, Gs, and Pn varied in opposite directions due to the increasing catalytic activity of antioxidant enzymes and the high Na^+^/K^+^ ratio. Moreover, the increasing activity of antioxidant enzymes SOD, POD, and CAT resulted in the suppression in the generation of ROS as indicated by the negative correlation between enzymatic activity and ROS. Overall, physiological traits (Chl, Pn, and Gs), osmolytes (proline and GB), and plant water relations (RWC) illustrated a positive correlation with respect to each other, while they illustrated a negative correlation with respect to antioxidant enzymes and Na^+^/K^+^ ratio. The correlation of traits varied significantly among all soybean cultivars as indicated by the PCA biplot. The scatter plot for genotypes confirmed the varying extent and type of association among physiological, biochemical, and water-related traits in all soybean genotypes as indicated by the varying length and spacing of the traits’ vectors with respect to the origin (**Figure 6**). In the PCA biplot, the genotypes G4620RX, DM45X61, and NARC-21 closely spaced to the traits’ vectors, indicating the strong association of traits among these genotypes. Conversely, the genotypes Rawal-1, NARC-1, and Swat-84 positioned away from the traits’ vectors in the biplot indicated the weak association of traits among these genotypes. Moreover, the slightly different distribution of eclipses with respect to the origin of the biplot also indicated the varying impact of genotypes on the association of traits. On the other hand, the scattered plot also confirmed the varying impact of individual and combined treatment of drought and salinity on the association of traits, as confirmed by the varying orientation of the traits’ vectors with respect to the origin (**Figure 7**). Besides that, the varying positioning of eclipse indicating combined drought and salinity from the biplot origin confirmed the varying effect of combined stress on the association of physiological, biochemical, and water-related traits compared with individual and control treatments. The heatmap dendrogram has further confirmed the results from biplots (**Figure 8**) and revealed a strong expression of physiological, biochemical, and water-related traits in soybean genotypes G4620RX, DM45X61, and NARC-21 compared with Rawal-1, NARC-1, and Swat-84 under control, individual, and combined treatments of stresses. Moreover, the different band colors of the dendrogram indicated the varying extent of the expression of each trait in soybean under different treatments.

### 3.6 Gene expression

**Figure 9:**
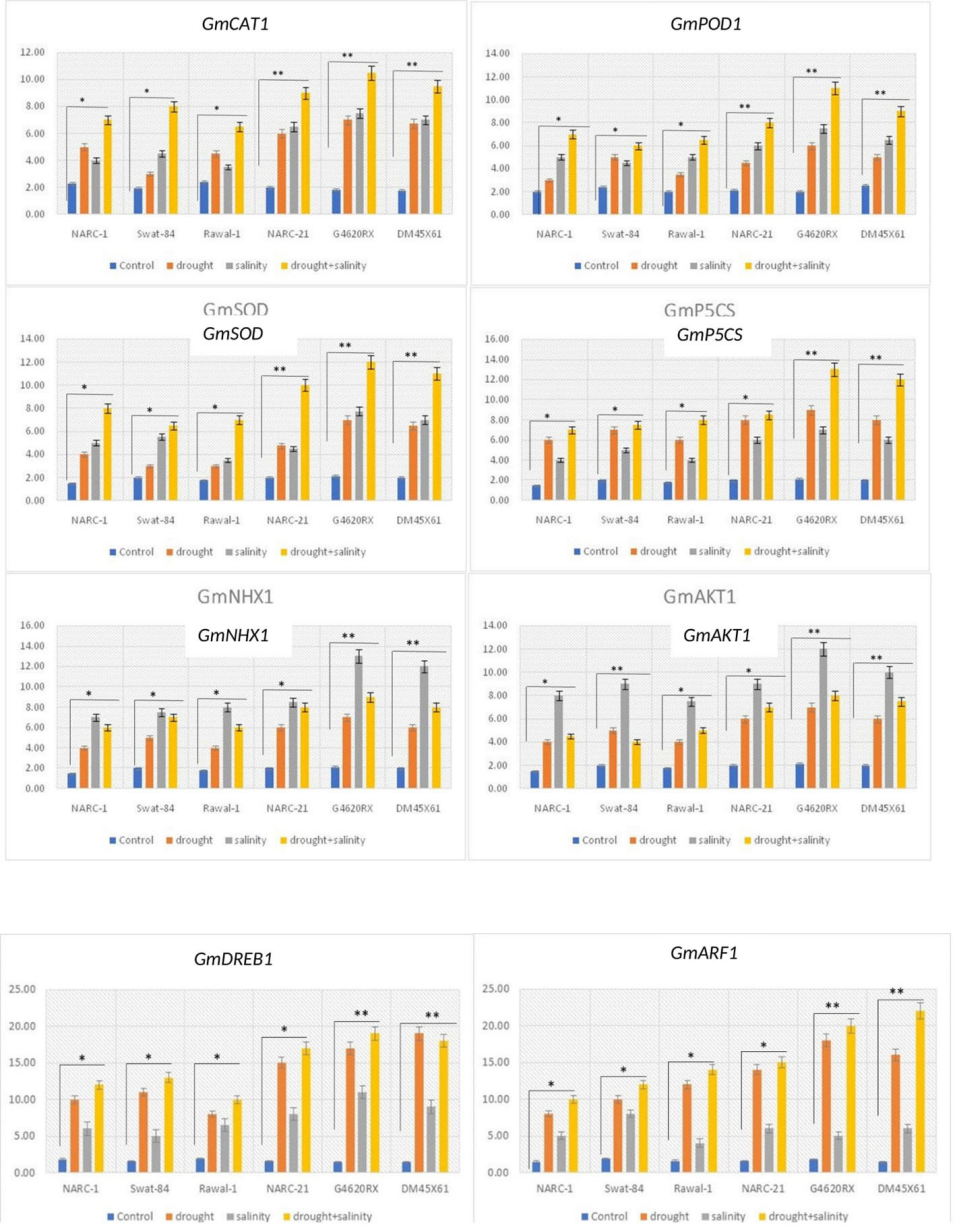
Relative expression of genes related to drought and salt stress tolerance in different soybean genotypes under individual and combined applications of drought and salt stress. **, significant at *p* ≤ 0.01; *, significant at *p* ≤ 0.05.

The genes *GmCAT1*, *GmPOD1*, and *GmSOD* illustrated a significant (*p* ≤ 0.05) variation in their expression in all soybean genotypes under individual and combined applications of drought and salinity stress (**Figure 9**). Under all versions of stresses, these genes showed a significantly (*p* ≤ 0.05) high level of transcripts compared with the control treatment, with a maximum transcript level at the combined application of stresses. Besides that, among genotypes, G4620RX, DM45X61, and NARC-21 illustrated a significantly high upregulation while the genotypes Rawal-1, NARC-1, and Swat-84 showed a significantly less upregulation of *GmCAT1*, *GmPOD1*, and *GmSOD1*. Similarly, the activity of antioxidant enzymes CAT, POD, and SOD was consistent with the regulation of these genes under corresponding individual and combined treatments of drought and salinity stress. The expression of *GmP5CS* gene varied significantly (*p* ≤ 0.05) in all soybean genotypes parallel to the accumulation of proline under individual and combined applications of drought and salinity treatments (**Figure 9**). Moreover, *GmP5CS* showed a significantly (*p* ≤ 0.05) high upregulation in all genotypes due to individual and integrated versions of drought and salinity treatments, with the highest upregulation under integrated treatment of stress. Furthermore, the genotypes G4620RX, DM45X61, and NARC-21 depicted a comparatively high increase while Swat-84, Rawal-1, and NARC-1 depicted a comparatively less increase in the expression of *GmP5CS*. The genes *GmAKT1* and *GmNHX1* recorded a significantly high level of transcripts in all soybean genotypes under individual and combined applications of drought and salinity compared with the control treatment (**Figure 9**). Unlike other genes, *GmAKT1* and *GmNHX*1 illustrated a significantly (*p* ≤ 0.05) high expression under saline condition compared with drought and combined drought and salinity stress. Among genotypes, Rawal-1, followed by NARC-1 and Swat-84, showed less upregulation, while DM45X61, followed by G4620RX and NARC-21, showed high upregulation of *GmAKT1* and *GnNHX1*. The high expression of these genes in soybean genotypes was in line with the decrease in Na^+^/K^+^ as these genes trigger the influx of K^+^. On the other hand, the expression of drought-responsive genes *GmDREB1* and *GmARF1* increased significantly in all soybean genotypes at drought and combined applications of drought and salt stress compared with the control and saline treatments (**Figure 9**). The genotype DM45X61, followed by G4620RX and NARC-21, illustrated a comparatively high increase in transcripts of *GmDREB1* and *GmARF1*, while the genotypes Swat-84, NARC-1, and Rawal-1 recorded a lesser increase in the transcripts of *GmDREB1* and *GmARF1*.

## 4 Discussion

Soil drought and salinity are potential stresses restricting plant productivity. The impacts of drought and salinity on plants ranges from morpho-physiological adaptations to biochemical and molecular responses. The responses of plants to the twin abiotic stresses are a bit unique such that they are difficult to predict in isolation. The initial responses of plants to drought and salt stress are primarily similar as both perturb physiological processes and osmotic balances. The twin drought and salt stress directly affect Pn, Gs, and chl due to biochemical perturbances such that they produce intense secondary oxidative stress compared with the isolated application of stress. In short, combined drought and salt stress causes additional adverse effects on plants’ physiological and osmotic traits. Therefore, all soybean cultivars showed more drop in Pn, Gs, chl, RWC, and water potential and more rise in proline, GB, O_2_^•−^, H_2_O_2_, and antioxidant activities under the twin application of compared with the sole application of drought and salt stress. During stress, the enhanced levels of ROS show signaling function in addition to the synthesis of antioxidant enzymes. Plants are naturally equipped with protective responses including stomatal closure, activating ROS scavenging, stopping photosynthesis, and activating the expression of stress-related genes. Numerous research studies have found that coupled water and salt stress has more adverse effects on plants (Angon et al., 2022; Cao et al., 2023; Fu et al., 2023). Besides that, high Na^+^/K^+^ ratio was recorded at salt and combined drought–salt stress, illustrating that water deficiency does not increase the deposition of Na in plants. Moreover, plant experiences more oxidative stress under combined drought–salinity stress compared with the isolated application of stresses as indicated by the high production of O_2_^•−^ and H_2_O_2_. Correspondingly, the high activities of antioxidant enzymes were recorded at the twin application of stress compared with sole stresses probably due to ROS scavenging mechanisms triggered due to the overproduction of ROS. In addition, the genes controlling the biochemical traits of stress tolerance were regulated differently under the sole and coupled applications of drought and salt stress. Overall, the integrated application of drought and salt stress depicted a high level of change in all physiological, biochemical, and indicators of stress tolerance.

Plants face oxidative stress due to the generation of reactive oxygen species such as hydrogen peroxide (H_2_O_2_) and superoxide radical (O_2_^•−^), which disrupts the structural integrity of membranes present in different organelles (Shah et al., 2017; Dos-Santos et al., 2022; Juan et al., 2021; Sachdev et al., 2021; Zhang et al., 2022). The abiotic stresses partially or wholly impede the vital physiological processes, including Gs and Pn, that are essential for plant survival (Ding et al., 2024). The coupled drought–salinity stress has an additional adverse effect on chlorophyll compared with individual stress, hence causing a high reduction in Pn and Gs compared to individual stress as reported by Otie et al. (2021) and Zhou et al. (2022) in soybean under salt and water stress, respectively. Similarly, the present study found a substantial reduction in chl, Gs, and Pn under combined drought–salt stress compared with individual stresses in all soybean genotypes (**Figure 1**). Generally, the levels of compatible solutes glycine betaine and proline increase additionally when the plant is exposed to multiple stresses (Wani et al., 2013). The compatible solutes are non-toxic at a high concentration and serve as osmoprotectant to protect the plant from multiple stresses in different ways, such as cellular osmotic adjustment, retention of membrane integrity, and detoxification of ROS as reported by Xu et al. (2023). **Figure 2** likewise illustrates the dynamic rise in the concentration of proline and GB under twin drought–salt stress in all soybean genotypes. Furthermore, the high proline and GB contents in soybean genotypes G4620RX, DM45X61, and NARC-21 compared with those in Rawal-1, NARC-1, and Swat-84 under coupled and individual drought and salinity treatment were an indicator of their strong molecular mechanism to counter the stress (**Figure 2**). Moreover, the leakage of K^+^ and influx of Na^+^ is possibly due to ROS production under water-deficit and salt-elevated conditions as confirmed through various studies (Feng et al., 2023; Ali and Rab, 2017; Demidchik and Maathuis, 2007; Wu, 2018). Besides that, salinity triggers the influx of Na^+^ and leakage of K^+^ that lead toward a high Na^+^/K^+^ ratio causing leaf necrosis, pigment degradation, and disruption of water and osmotic potentials (Otie et al., 2021). Parallel with these findings, the current study reported a significant increase in Na^+^/K^+^ in all soybean genotypes due to saline and drought conditions (**Figure 2**). Conversely, the soybean genotypes G4620RX, DM45X61, and NARC-21 illustrated a lesser increase in Na^+^/K^+^ ratio even under saline stress, which is attributed to their high salt tolerance and speedy influx of K^+^ as explained by Ali and Rab (2017). Furthermore, plants possess various enzymatic and non-enzymatic processes to detoxify the harmful effect of oxidative stress imposed by ROS produced as a consequence of sole and combined drought–salinity stress (Liu et al., 2017; Hasanuzzaman et al., 2020). As the coupled drought–salt stress poses an additional oxidative stress, it therefore necessitates a comparatively high catalytic activity of antioxidant enzymes as reviewed by Cao et al. (2023). Hence, an increase in the catalytic activity of antioxidant enzymes CAT, SOD, and POD indicates the activation of the ROS scavenging mechanism (Rajput et al., 2021; Kesawat et al., 2023). The reduction in H_2_O_2_ and superoxide (O_2_^•−^) concentration owing to the enhanced activity of antioxidant enzymes is an indicator of tolerance to oxidative stress as reported by Khan et al. (2009) and Liu et al. (2017) in soybean. Similarly, in the present study, the soybean genotypes G4620RX, DM45X61, and NARC-21 manifested a high activity of SOD, POD, and CAT for ROS (H_2_O_2_ and O_2_^•−^) scavenging under coupled drought–salt stress compared with their individual application (**Figure 3**). This proves the high biochemical tolerance of these genotypes compared with Rawal-1, NARC-1, and Swat-84. Under abiotic stresses, plants can likewise alter water relation in order to retain various cellular functions (Zhang et al., 2023)—for instance, plants undergo osmotic adjustment through the accumulation of compatible osmolytes such as glycine betaine (GB) and proline (Doğan, 2011; Akitha et al., 2015; Ghosh et al., 2021). These osmotic adjustments intimate the plant to keep the cell volume at low water potential (ψ_w_), which is vital to maintain the metabolic functions (Ghosh et al., 2021). Besides that, the high RWC, increasing osmotic potential (ψ_s_), and decreasing water potential (ψ_w_) under drought and salinity stress illustrate the crop’s high tolerance to drought and salinity stress as reported by Wijewardana et al. (2019) and Otie et al. (2021), respectively, in soybean. The present study has further confirmed their findings and reported high RWC, high osmotic potential (ψ_s_), and low water potential (ψ_w_) in soybean genotypes G4620RX, DM45X61, and NARC-21, exhibiting comparatively high physiological and biochemical tolerance under drought and salinity stress (**Figure 4**). In plants, the physiological and biochemical traits are strongly associated, and their correlation varies according to the type of genotype and nature of stress (Liu et al., 2017; Vital et al., 2022; Ding et al., 2024). Moreover, the extent of physiological and biochemical responses under drought and salinity varies due to the varying tolerance tendency of soybean genotypes as shown in **Figures 5**–**7**. Besides that, the heatmap analysis has further confirmed the varying intensity of expression of each trait in all soybean genotypes under individual and integrated levels of drought and salinity stress (**Figure 8**). To date, various studies have been conducted to investigate the impacts of drought and salinity on the physiological and biochemical processes of soybean, but studies investigating the impacts on oxidative stress, physio-chemical changes, osmolytic dynamics, and genetic indices are limited. Although stress tolerance mechanisms vary from crop to crop, basic cellular responses to abiotic stresses are almost conserved in most of the plant species (Zhang et al., 2023)—for example, different abiotic stress elements trigger oxidative stress, protein denaturation, and osmotic stress in plants, which result in identical adaptive strategies such as production of stress proteins, induction of ROS-scavenging mechanisms, and accumulation of compatible osmolytes (Sachdev et al., 2021). Therefore, it is necessary to understand the physiological and biochemical markers of stresses with respect to their genetic determinants. Besides that, the genes *GmCAT1*, *GmSOD*, and *GmPOD*1 detoxify ROS through regulating the activities of antioxidant enzymes CAT, SOD, and POD, respectively. The antioxidant enzyme SOD converts superoxide (O_2_^•−^) to H_2_O_2_, which is further detoxified to O_2_ and H_2_O due to the catalytic activities of CAT and POD (Wang et al., 2018). Li et al. (2020) noticed the higher transcript of *GmCAT1*, *GmSOD*, and *GmPOD1* in tolerant soybean genotypes under oxidative stress imposed by high aluminum content that substantially lowered the concentration of ROS, including H_2_O_2_ and superoxide radical (O_2_^•−^). Correspondingly, the current study reported the significant upregulation of *GmCAT1*, *GmPOD1*, and *GmSOD* in soybean genotypes G4620RX, DM45X61, and NARC-21 along with the increased catalytic activities of antioxidant enzymes CAT, POD, and SOD that caused a significant decline in ROS (H_2_O_2_ and O_2_^•−^) under oxidative stress imposed by sole and twin drought and salt stress (**Figure 9**). This has confirmed the potential role of genetic determinants in regulating the antioxidant potential of soybean genotypes through regulating the biochemical mechanisms. Xu et al. (2023) confirmed that *GmP5CS* is a downstream gene of *GmCOL1a* whose overexpression improves the salt tolerance and drought resistance in soybean due to an increase in proline content, RWC, and CAT, POD, and SOD catalytic activities. Similar to these findings, the current study recorded a parallel increase in the expression of *GmP5CS* along with RWC, proline concentration, and enzymatic (SOD, POD, and CAT) activities in soybean genotypes G4620RX, DM45X61, and NARC-21, showing more physiological and biochemical tolerance under isolated and combined versions of drought and salinity stresses (**Figure 9**). Furthermore, Jin et al. (2022) and Sun et al. (2021) found that NHX gene improves the salt tolerance tendency of soybean by decreasing the Na^+^ content in the cytoplasm or by keeping a low Na^+^/K^+^ ratio. Besides that, Sun et al. (2019) has further confirmed that, in addition to mediating the efflux of Na^+^, *GmNHX1* regulates the expression of a series of other genes, including *SKOR*, *SOS1*, and *AKT1*, involved in salinity tolerance. Moreover, Wang et al. (2021) reported the essential role of *GmAKT1* in the uptake of K^+^ to balance the Na^+^/K^+^ ratio under saline conditions. Complementary to these findings, the current study has recorded a significant increase in the uptake of K due to the increased expression of *GmNHX*1 and *GmAKT1* in the genotypes G4620RX, DM45X61, and NARC-21 as evidenced by their decreased Na^+^/K^+^ ratio compared with Rawal-1, Swat-84, and NARC-1 (**Figure 9**). In fact, the molecular crosstalk of *GmAKT1* with plant phytohormones triggers the essential physiological mechanisms providing soybean with tolerance against abiotic stress (Wang et al., 2021). The overexpression of *GmDREB1* confers soybean with tolerance against drought stress as reported by Zhou et al. (2022) and Chen et al. (2022). The current study likewise recorded a consistent increase in the expression of *GmDREB1* in soybean cultivars G4620RX, DM45X61, and NARC-21, showing high physiological and biochemical tolerance to drought and salt stress (**Figure 9**). This can be attributed to the tendency of *GmDREB1* to regulate the expression of osmotic and oxidative stress-related protein that reduces the cell injury caused by salt and drought stress, thus improving the stress tolerance of soybean (Jiang et al., 2014; Kidokoro et al., 2015). Besides that, Ha et al. (2015) recorded the potential role of *GmARFs* in inducing drought tolerance in soybean through modulating the interaction of auxins with other hormones. Correspondingly, the current study confirmed their findings and found a dynamically increasing expression of *GmARF1* in genotypes G4620RX, DM45X61, and NARC-21 showing physiologically and biochemically high tolerance against drought stress (**Figure 9**).

## 5 Conclusion

In the present study, combined drought and salt stress altered soybean’s physiological, biochemical, genetic, and osmotic responses more intensively rather than their individual application. This proves that simultaneous exposure of plants to multiple abiotic stresses causes a severe manipulation in plant traits; however, the extent of plant trait manipulation strictly adheres with the plant’s ability to withstand stress. Furthermore, the present study also revealed that tolerance to abiotic stresses is undoubtedly an intricate phenomenon at the cellular and whole plant levels. In fact, this is due to the complex interaction between stress elements and different physiochemical and molecular mechanisms determining the plant’s growth and developmental processes. Overall, the soybean genotypes G4620RX, DM45X61, and NARC-21 depicted a high tolerance to the combined and individual applications of salt and drought stresses based upon physiological and molecular mechanisms. As the development of stress-tolerant crops requires knowledge on the contributing physiological and biochemical processes and the genetic control of participating traits, the present study will hence provide a considerable insight to elucidate the molecular dynamics of abiotic stress tolerance in soybean. Besides that, it will further contribute in devising soybean breeding strategies against drought and salt stresses by focusing on physio-chemical and genetic dynamics in unison.

## Data Availability

The original contributions presented in the study are included in the article/supplementary material. Further inquiries can be directed to the corresponding author.
